# Hooked on a feeling: affective anti-smoking messages are more effective than cognitive messages at changing implicit evaluations of smoking

**DOI:** 10.3389/fpsyg.2015.01488

**Published:** 2015-10-06

**Authors:** Colin Tucker Smith, Jan De Houwer

**Affiliations:** ^1^Department of Psychology, University of FloridaGainesville, FL, USA; ^2^Ghent UniversityGhent, Belgium

**Keywords:** implicit evaluations, affect, persuasion, smoking, substance use

## Abstract

Because implicit evaluations are thought to underlie many aspects of behavior, researchers have started looking for ways to change them. We examine whether and when persuasive messages alter strongly held implicit evaluations of smoking. In smokers, an affective anti-smoking message led to more negative implicit evaluations on four different implicit measures as compared to a cognitive anti-smoking message which seemed to backfire. Additional analyses suggested that the observed effects were mediated by the feelings and emotions raised by the messages. In non-smokers, both the affective and cognitive message engendered slightly more negative implicit evaluations. We conclude that persuasive messages change implicit evaluations in a way that depends on properties of the message and of the participant. Thus, our data open new avenues for research directed at tailoring persuasive messages to change implicit evaluations.

## Introduction

As Zajonc (1980) pointed out in his classic paper, people often respond to stimuli in an evaluative manner even when they do not have the intention to do so, when they have the intention not to do so, or when they are unaware of the stimuli, the reaction, or the link between both. Such automatic evaluative responses – or “implicit evaluations” as we will call them (De Houwer, 2009; De Houwer et al., 2013) – have proved to be a fecund research topic. Over the past two decades, researchers have explored their properties, developed ways to measure them, and examined their impact on behavior (see Ferguson and Zayas, 2009; De Houwer and Hermans, 2010, for reviews). Importantly, numerous studies indicate that the use of implicit measures contributes in a significant manner to our understanding of psychological phenomena in such varied domains as addiction and substance use (Wiers and Stacy, 2006; Rooke et al., 2008), consumer behavior (Friese et al., 2006), psychopathology (see Roefs et al., 2011), and social interactions (Fazio and Olson, 2003). Indeed, implicit measures indicate unique predictive validity even when one takes into account direct self-report (Greenwald et al., 2009).

Given the importance of implicit measures in predicting behavior, researchers have been consistently interested in how to influence them. Theoretical views on the potential to change implicit evaluations quickly evolved from what could be characterized as a “slow to build and slow to change” position (e.g., Smith and DeCoster, 1999; Wilson et al., 2000; Rydell and McConnell, 2006) to a stance that implicit evaluations are “fast to build, but slow to change” (Gregg et al., 2006). Now, as evidence continues to accumulate regarding ways in which implicit measures are amenable to experimental manipulations (for reviews see Blair, 2002; Gawronski and Sritharan, 2010), we seem to be nearing the “fast to build and fast to change” stage. Although there is increasing acceptance of the idea that implicit measures are quite malleable, this is not a “yes or no” question. Instead, it is important to know when and how this malleability occurs. Learning more about the conditions under which implicit measures can be influenced arguably adds to our understanding of implicit evaluations and helps us to build more efficient and effective interventions for changing behavior by changing implicit evaluations.

Before discussing our research, we present a brief overview of prior research and theorizing about changes in implicit evaluations. Gawronski and Bodenhausen (2011) argued that there are two primary processes by which implicit evaluations can change – altering the structure of associations in memory that give rise to those evaluations or influencing the activation level of existing associations. It is typically assumed that the structure of associations can be changed only gradually through repeated experiences. For example, implicit evaluations of stimuli might be changed by repeatedly pairing them with other positive/negative stimuli (e.g., De Houwer et al., 1998; Baccus et al., 2004; Dijksterhuis, 2004) or positive/negative actions such as repeatedly pulling a joystick toward oneself in response to images of Black people (e.g., Kawakami et al., 2007). The second route to shifting implicit evaluations (i.e., influencing the activation of associations) is most often studied by changing the context of evaluation. For example, implicit race bias is reduced after Black and White faces are viewed in the context of a church as compared to a graffiti-covered street corner (Wittenbrink et al., 2001). Context changes are also achieved by presenting pictures of counter-stereotypical exemplars (e.g., Dasgupta and Greenwald, 2001) or by having participants imagine such exemplars (Blair et al., 2001). Implicit bias has also been reduced by changing the evaluative context through stimulus recategorization. For instance, implicit race bias decreases when participants categorize liked Black athletes and disliked White politicians on the basis of their profession rather than their race (Mitchell et al., 2003). Each of these context variables is thought to affect implicit evaluations by influencing what knowledge content is activated in memory (e.g., existing associations between Black people and positive).

Most studies on changes in implicit evaluations have used either extensive training (which is assumed to capitalize on the first route) or context manipulations (which is assumed to capitalize on the second route) as instruments for change. Recently, however, it has been demonstrated that persuasive verbal messages can also lead to changes in implicit evaluations. For instance, Horcajo et al. (2010) found that implicit preferences for vegetables relative to animals could be increased by providing participants with a persuasive argument about positive aspects of eating vegetables. Like other changes in implicit evaluations, these effects might be produced either by activating existing knowledge structures or by adding new knowledge to memory. The latter idea is rarely considered because many theories postulate that implicit evaluations are the product of associations that develop gradually as the result of many experiences (e.g., Rydell and McConnell, 2006). Based on this assumption, one would expect that extensive retraining is necessary to change associations – and thus implicit evaluations – in a lasting manner. Single events such as the presentation of a persuasive message should not have this effect. However, recent evidence suggests that verbal messages can change implicit evaluations by adding information to memory. For instance, simply telling participants that one (fictitious) social group has more positive traits than another (fictitious) social group is sufficient to see more positive implicit evaluations of the former group (De Houwer, 2006; Gregg et al., 2006; Ranganath and Nosek, 2008). Such effects cannot be due to the impact of the verbal message on the activation of existing knowledge structures because the social groups were new to the participants. On the one hand, this insight blurs the seemingly neat divide between implicit and explicit cognition. It seems likely that the complexity of this distinction has been greatly underestimated in the past (see De Houwer et al., 2009; De Houwer, 2014). On the other hand, it points at interesting novel routes for changing implicit evaluations. More specifically, if verbal messages can have effects on novel implicit evaluations, perhaps persuasive messages can result in changes in the content of knowledge that produces implicit evaluations toward well-established attitude objects (see Hughes et al., 2011, for a theoretical account). Because it is often easier to expose people to persuasive messages than to extensive training programs, persuasion of implicit evaluations might turn out to be both an effective and efficient way of inducing relatively stable changes in implicit evaluations. Future research on the persuasion of implicit evaluations might not only have applied value, but could also lead to a more realistic appreciation of the complex relation between implicit and explicit cognition.

In order to evaluate the potential of persuasion of implicit evaluations, however, it does not suffice to examine *whether* it can take place. We also need to learn more about *when* it occurs and how its effects can be maximized. Although it is now clear that persuasive messages can lead to changes in implicit evaluations, little is yet known about the moderators of these changes. We recently showed that a persuasive message about a consumer item is more impactful on implicit evaluations when attributed to a source high in credibility (i.e., expertise and trustworthiness) as compared to low in credibility (Smith et al., 2013). Similar effects based on a source’s likeability and attractiveness were observed using the Implicit Association Test (IAT; Smith and De Houwer, 2014). Relatedly, people show an increased preference for a political proposal when presented by a member of their political ingroup (Smith et al., 2012). In each of those investigations, all participants read an identical message and the *source* of the message was manipulated; in the present work, we focus on another crucial aspect of persuasive messages, namely the *content* of the messages.

From the range of content-related dimensions that might moderate the impact of persuasive messages on implicit evaluations, we decided to begin with the affective or cognitive nature of the message for two reasons. First, prior research has shown that the affective or cognitive nature of persuasive messages is an important moderator in the persuasion of explicit evaluations. More specifically, affective arguments are more effective at changing evaluations that are affective in nature whereas cognitive arguments are more effective at changing evaluations that are cognitive in nature (e.g., Fabrigar and Petty, 1999). Given that research has revealed important parallels between the variables that moderate persuasion of explicit evaluations and those that moderate persuasion of implicit evaluations (e.g., Marini et al., 2012; Smith et al., 2013), one can predict that the affective or cognitive nature of the persuasive message could have a similar impact in the persuasion of implicit evaluations. That is, those implicit evaluations that are primarily affective in nature might be more susceptible to the impact of affective than cognitive persuasive messages whereas the reverse might be true for those implicit evaluations that are primarily cognitive in nature.

In our studies, we focused on implicit evaluations of smoking in smokers in part because there are good reasons to assume that these evaluations are mostly affective in nature. Hence, based on the general principle that persuasive messages have more impact when they match the targeted evaluations in terms of the affective or cognitive nature, we can be fairly confident in our prediction that implicit evaluations of smoking in smokers should change more effectively in line with affective persuasive messages than cognitive persuasive messages. The reasons we believe that implicit evaluations of smokers are affective in nature are twofold. First, a number of findings suggest that implicit evaluations are generally more affect-based than explicit evaluations. For instance, a meta-analysis found the relationship between implicit and explicit evaluations is strengthened when explicit evaluations are more affective as opposed to cognitive (Hofmann et al., 2005; see Gawronski and LeBel, 2008; Smith and Nosek, 2011 for additional evidence for the relatively affective nature of implicit evaluations). Second, there is evidence that explicit evaluations of smoking in smokers are more related to affect than to cognition (Trafimow and Sheeran, 1998). If smokers’ explicit evaluations of smoking tend to be affect-based and implicit evaluations generally tend to be more affect-based than the explicit evaluations, it is very likely that implicit evaluations of smoking are affect-based.

In the current work, we operationalized affect and cognition through the use of anti-smoking persuasive messages that have been shown to effectively appeal to either *feelings* or *reasoning* (See et al., 2008). In four studies, we presented participants with persuasive anti-smoking messages focusing on affective or cognitive arguments. We compared the relative effectiveness of these two types of verbal persuasive messages at changing implicit evaluations of smoking among current smokers. The focus on implicit evaluations of smoking in current smokers has the additional advantage that it provides a strong test of the power of persuasive messages because implicit evaluations toward smoking are likely to be long-standing, important evaluations.

In Studies 1 and 2 we test the prediction that implicit evaluations of smoking in smokers will be more negative after reading an affective anti-smoking message as compared to a cognitive anti-smoking message. In these studies, implicit evaluations are captured using a personalized version of the IAT (Olson and Fazio, 2004), an Affect Misattribution Procedure (AMP; Payne et al., 2005), and an Evaluative Priming task (EP: Fazio et al., 1986). In Study 3, we include a control condition and test predictions using the IAT (Greenwald et al., 1998), in addition to also testing effects among non-smokers. Finally, in a fourth study using the AMP, we again include a control condition and test the mediational process by which the anti-smoking messages impact implicit evaluations. Although the focus of the current work is on the moderation and mediation of changes in implicit evaluations, we also include explicit evaluations in all four studies in the interest of completeness and comparison. Participants in all studies read a consent form and were debriefed about the purpose of the study; deception was not utilized in any study, and all studies were conducted in line with the ethical guidelines of Ghent University.

## Study 1

### Method

#### Participants

Participants were 220^1^ visitors to the Project Implicit research site who self-reported that they currently smoked cigarettes; 66.1% women (*M*_age_ = 30.5; S*D* = 10.2; range: 18–69). Participants were citizens of 29 different countries (71.8% USA, 6.4% Canada, 5.5% UK, all others < 1.5%). The modal response for smoking behavior was “I smoke between 1 and 5 cigarettes per day.”

#### Materials

##### Persuasive messages

Participants read one of two brief anti-smoking messages that was either affective or cognitive in content. Previous research by See et al. (2008) showed the affective message is successful at eliciting relatively more feelings than thoughts while the cognitive message elicits relatively more thoughts than feelings. However, it is important to note that although See et al. (2008) established that these persuasive messages differ on engendering feelings or thoughts, they did not test how persuasive the messages were (neither in terms of their impact on explicit evaluations nor in terms of perceived persuasiveness). Therefore, we asked 38 self-reported smokers to evaluate the strength of the anti-smoking message from “not at all strong” to “extremely strong” with higher scores indicating greater perceived argument strength. Participants did not report a difference in perceived strength of the arguments based on whether the argument was affective (*M* = 3.00, *SD* = 1.33) or cognitive (*M* = 2.95, *SD* = 0.89), *t*(36) = 0.14, *p* = 0.89, *d* = 0.04.

##### Personalized IAT

Participants completed a variant of the IAT designed to measure the strength of associations between “smoking,” “non-smoking,” “I like,” and “I dislike” (see Han et al., 2006, Experiment 2, for a similar approach). Participants sorted stimuli quickly while making as few errors as possible. Category labels appeared in the upper-left and upper-right of the screen and participants used the “E” key and “I” key to sort stimuli to the left and right, respectively. Stimuli for smoking and non-smoking categories were six pictures of smoking-related stimuli (e.g., cigarette, man smoking) and six images related to “not smoking” (e.g., man blowing a whistle, pencil being sharpened), while stimuli for evaluative categories were positive and negative words. The number and order of trials in the IAT followed the recommendations of Nosek et al. (2005). IAT scores were calculated using the *D*-algorithm (Greenwald et al., 2003) with positive numbers indicating a preference for smoking relative to not-smoking. Data from 16 participants (7.3%) were deleted for making too many errors (>40% in any one block or >30% overall); split-half correlation of the remaining 204 IAT scores was *r* = 0.64.

##### Affect Misattribution Procedure

In each trial of the AMP, participants viewed a prime stimulus followed by a Chinese pictograph. Participants were instructed to ignore the initial stimulus and rate whether the pictograph was more pleasant or less pleasant than average. Previous research has shown the affective valence of the prime can bleed over into ratings of the Chinese pictographs. In the current study, participants viewed three types of primes; the images used in the IAT were used for “smoking” and “non-smoking” primes and a gray rectangle served as a neutral prime. Participants saw 24 of each of the three types of primes, for a total of 72 trials. An individual trial began with the presentation of the prime (100 ms). This was followed by a blank screen (100 ms) after which one of the 72 Chinese pictographs was presented (100 ms). Finally, a black and white mask image was presented until the participant responded. This procedure produces indicator of implicit positivity for each of the three types of trials by calculating the proportion of “pleasant” responses following each of the categories of primes (i.e., smoking, not-smoking, and neutral). In addition, individual AMP scores can be calculated by subtracting the proportion of positive responses following non-smoking responses from those following smoking responses. In this way, positive scores indicate a relative implicit preference for smoking. Data from three participants (1.4%) who responded either “positive” or “negative” to all 72 trials were deleted; split-half reliability of the remaining 217 AMP scores was *r* = 0.59.

##### Explicit evaluations

Participants reported their evaluations of smoking by responding to “Which of the following statements best describes you?” using a seven-point response scale ranging from -3 to +3, anchored by “I strongly prefer smoking to not smoking” and “I strongly prefer not smoking to smoking.” Positive scores indicate a preference for smoking.

#### Procedure

Visitors to the Project Implicit research site first register to be involved in research studies at which time they complete demographic information. After completing informed consent procedures, participants were asked to indicate whether they smoked cigarettes. Those participants who said “no” were then reassigned to complete a different study within the Project Implicit research pool. Participants who responded “yes” were asked to report how much they smoked. They were then told the study regarded reading and memory, were randomly assigned to read the affective or cognitive persuasive message, and completed the personalized IAT, AMP, and explicit preferences in that order.

### Results

Overall, participants indicated strong implicit preferences for non-smoking relative to smoking using the personalized IAT, *M* = -0.33, *SD* = 0.52, *t*(203) = -8.99, *p* < 0.0001, Cohen’s *d* = -0.63 and the AMP, *M* = -0.18, *SD* = 0.32, *t*(216) = -8.09, *p* < 0.0001, *d* = -0.55. Participants’ self-reported preference for smoking did not differ from zero (i.e., no preference), *M* = 0.01, *SD* = 1.98, *t*(217) = 0.10, *p* = 0.91, *d* = 0.01. Participants’ self-reported preferences correlated with the personalized IAT at *r* = 0.21, *p* = 0.002 and with the AMP at *r* = 0.21, *p* = 0.002; scores on the personalized IAT correlated with AMP scores at *r* = 0.21, *p* = 0.004.

#### Explicit Evaluations

Smokers’ self-reported evaluations were unaffected by the type of persuasive message they read; evaluations were not more negative following the affective message (*M* = -0.02, *SD* = 2.01) than the cognitive message (*M* = 0.07, *SD* = 1.94), *t*(216) = -0.33, *p* = 0.74, *d* = 0.05.

#### Personalized IAT

When measured using the personalized IAT, smokers’ implicit evaluations were not more negative following the affective message (*M* = -0.34, *SD* = 0.52) than the cognitive message (*M* = -0.30, *SD* = 0.51), *t*(202) = 0.52, *p* = 0.60, *d* = 0.08, 95% CI_diff_ = -0.45, 0.63.

#### Affect Misattribution Procedure

We tested the effect of the persuasive messages on the AMP by comparing the difference scores composed of responses to the smoking and non-smoking trials of the AMP. AMP scores were significantly affected by the type of persuasive message they read; evaluations were more negative following the affective message (*M* = -0.24, *SD* = 0.37) than the cognitive message (*M* = -0.10, *SD* = 0.23), *t*(215) = 3.16, *p* = 0.002, *d* = 0.45, 95% CI_diff_ = 0.05, 0.22.

### Discussion

In the current study, we observed for the first time that implicit evaluations of smoking in smokers – as measured by the AMP – were more anti-smoking following an affective anti-smoking message than after a cognitive anti-smoking message. This observation confirms earlier findings showing that directly persuasive messages can change implicit evaluations, but also shows that the effect depends on the content of the persuasive message. In contrast, the content of the message did not have an impact on explicit evaluations. Finally, the content of the message did not have an effect on the second measure of implicit evaluations, the personalized IAT. Therefore, to test whether the observed effects are limited to the AMP, we ran a second study that again used the personalized IAT, but also included another implicit measure, namely the evaluative priming task.

## Study 2

### Method

#### Participants

Participants were 254 visitors to the Project Implicit research site who self-reported that they currently smoked cigarettes; 68.5% women (*M*_age_ = 33.2; *SD* = 11.9; range: 18–72). Participants were citizens of 32 different countries (69.2% USA, 7.1% Canada, 5.5% UK, all others < 2.5%). The modal response for smoking behavior was “I smoke between 10 and 20 cigarettes per day.”

#### Materials

##### Persuasive messages

Affective and cognitive messages were identical to those presented in Study 1.

##### Personalized IAT

The personalized IAT was identical to the one used in Study 1. Positive scores again indicate a relative preference for smoking. IAT scores from the 14 participants (7.3%) who committed too many errors were deleted; for the remaining 240 participants, split-half correlation of the IAT was *r* = 0.59.

##### Evaluative priming

In the EP task, participants were told that words and images would appear one after the other on the screen. They were instructed that their task was simply to classify each word they saw as being good or bad using the ‘E’ and ‘I’ keys of a computer keyboard. The labels “bad” and “good” appeared in the left and right upper corners of the screen, respectively. A single trial consisted of a fixation cross presented in white (500 ms), a blank screen (500 ms), a prime (200 ms), a post-prime pause (50 ms), and the presentation of a target word in white font (1500 ms). The inter-trial interval was set to vary randomly around 1000 ms with the limits of 500 and 1500 ms. There were four types of trials – smoking+good, smoking+bad, non-smoking+good, and non-smoking+bad. Participants first completed eight practice trials (two of each of the four types of trials), then completed 180 trials separated into three blocks of 60; each of the three blocks of 60 trials contained 15 of the four types of trials (e.g., smoking+good). The EP task was scored by dropping trials with an incorrect response (7.5%) as well any trials on which reaction times were at least 2.5 standard deviation removed from an individual’s mean for that type of trial (9.3% of all trials). Finally, data from participants who made errors at a rate >2.5 standard deviation from the mean was deleted (30 participants; 8% of potential data). A difference score was created for each participant by subtracting mean latencies for smoking+bad trials from mean latencies for smoking+good trials. A second difference score was created in the same way for non-smoking trials so that, in both cases, higher scores indicated positivity. Following this, a difference score was constructed by subtracting positivity toward Non-smoking from positivity toward Smoking. Thus, positive scores indicate a preference for smoking; split-half reliability of the EP scores was *r* = 0.31.

##### Explicit evaluations

Explicit evaluations were measured as in Study 1; positive scores again indicated a relative preference for smoking.

#### Procedure

The procedure followed that of Study 1 exactly, except for the replacement of the AMP with an EP task. Participants completed a personalized IAT and the EP task in a counterbalanced order and always reported explicit evaluations last.

### Results

Overall, participants indicated a strong implicit preference for non-smoking relative to smoking using the personalized IAT, *M* = -0.41, *SD* = 0.51, *t*(239) = -12.42, *p* < 0.0001, *d* = -0.80 and the EP task, *M* = -24.8, *SD* = 62.6, *t*(249) = -6.27, *p* < 0.0001, *d* = -0.40. Participants’ self-reported preference for smoking did not differ from zero (i.e., no preference), *M* = 0.21, *SD* = 1.97, *t*(251) = 1.70, *p* = 0.091, *d* = 0.11. Participants’ self-reported preferences correlated with the personalized IAT at *r* = 0.28, *p* < 0.0001, but did not correlate with the EP task, *r* = 0.003, *p* = 0.97; scores on the EP task, however, did correlate with scores on the personalized IAT, *r* = 0.21, *p* = 0.001.

#### Explicit Evaluations

Explicit preferences were unaffected by the manipulation of message type; evaluations were not more negative following the affective message (*M* = 0.15, *SD* = 2.01) than the cognitive message (*M* = 0.27, *SD* = 1.92), *t*(250) = 0.47, *p* = 0.64, *d* = -0.06, 95% CI_diff_ = -0.37, 0.61.

#### Personalized IAT

Confirming the hypothesis of this study, smokers’ implicit evaluations of smoking were more negative when measured using the personalized IAT following the affective message (*M* = -0.49, *SD* = 0.48) than the cognitive message (*M* = -0.32, *SD* = 0.53), *t*(238) = -2.60, *p* = 0.01, *d* = 0.33, 95% CI_diff_ = 0.04, 0.30.

#### Evaluative Priming

Smokers’ implicit evaluations of smoking were also more negative on the EP task following the affective message (*M* = -33.06, *SD* = 64.03) than the cognitive message (*M* = -16.32, *SD* = 60.12), *t*(248) = 2.13, *p* = 0.034, *d* = 0.27, 95% CI_diff_ = 1.26, 32.22.

### Discussion

In Study 2, using two very different implicit measures, we observed that implicit evaluations of smoking in smokers were more negative after reading an affective anti-smoking message than after reading a cognitive anti-smoking message. Explicit evaluations, again, were not differentially affected by the type of message. It should be noted that, whereas in Study 1 implicit evaluations captured by the personalized IAT were not more negative following the affective than cognitive message, in the current study they were (see LeBel and Paunonen, 2011, for a discussion of replication with implicit measures). As such, we conducted a combined analysis across the two studies; this test revealed a main effect of message type on the personalized IAT, *t*(442) = 2.09, *p* = 0.037, *d* = 0.20 which suggests that we can take seriously the differential impact of affective and cognitive messages on the personalized IAT.

Importantly, because both Studies 1 and 2 involved only a comparison of the effect of an affective vs. a cognitive negative message, we cannot yet make conclusions regarding the effectiveness of each type of message. In principle, both could have resulted in a more negative implicit evaluation, but the affective message more so than the cognitive message. However, it is also possible that the cognitive message resulted in less negative implicit evaluations of smoking (i.e., reactance) whereas the affective message might have had no effect. To examine this further, we next included a control condition to allow us to separately examine the effect of the affective and cognitive messages. In addition, we included non-smoking participants to examine whether the persuasive messages have a similar impact on smokers and non-smokers. Finally, we used a fourth implicit measure, the standard IAT.

## Study 3

### Method

#### Participants

Participants were 1055 visitors to the Project Implicit research website; 68.3% women (*M*_age_ = 29.2; *SD* = 11.4; range: 18–74). Participants were citizens of 69 different countries (79.3% USA, 3.6% UK, 3.2% Canada, all others < 1%). Of the 1039 who reported their smoking behavior, 17.8% were smokers, 18.6% former smokers, and 63.6% non-smokers. Only those participants reporting being current smokers (*n* = 185) or non-smokers (*n* = 723) were retained for analyses, leaving a usable sample of 908. For participants who reported being current smokers, the modal response for smoking behavior was “I smoke between 1 and 5 cigarettes per day.”

#### Materials

##### Anti-smoking messages

The affective and cognitive messages were identical to those used in Studies 1 and 2. In the control condition, participants read a negative review of an apartment complex.

##### Implicit Association Test

The IAT followed the same presentation and scoring procedure as in Studies 1 and 2 except that the labels “I like” and “I dislike” were changed to “Positive” and “Negative.” Positive scores again indicated a preference for smoking relative to not-smoking; split-half correlation of the IAT was *r* = 0.62.

##### Explicit evaluations

Participants reported their preference for smoking relative to non-smoking as in the two previous studies; positive numbers again indicate a preference for smoking.

#### Procedure

Participants randomly assigned to complete the current study were informed they were going to complete a study about reading comprehension and memory. Immediately following these instructions, participants were asked “Which of the following is most true for you?” with the answer choices of “I currently smoke cigarettes,” “I used to smoke cigarettes, but I quit,” and “I have never smoked cigarettes.” If they reported they currently smoke cigarettes, they were asked how much they currently smoke. Following this, participants were randomly assigned to read either the affective anti-smoking, cognitive anti-smoking, or control message. Immediately after the manipulation, participants completed the smoking IAT and explicit self-report in that order.

### Results

#### Implicit Association Test

**FIGURE 1:**
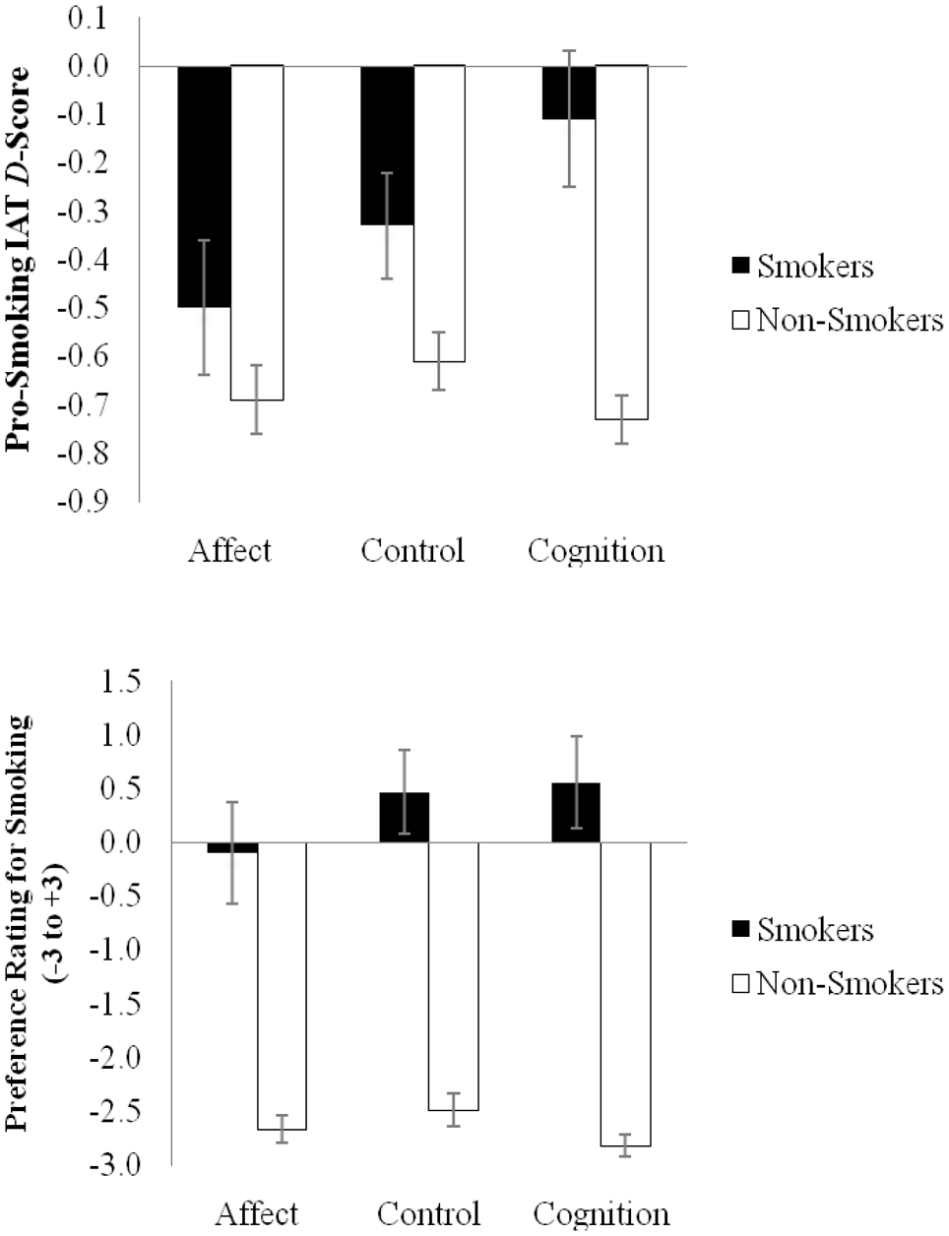
**IAT *D*-scores (top) and preference ratings (bottom) for smoking as a function of smoking status and the type of persuasive message in Study 3.** Positive scores indicate a preference for smoking. Error bars indicate the 95% confidence interval around the mean.

Hypotheses were tested using a 3(Condition: Affect vs. Cognition vs. Control) × 2(Smoking Status: Smoker vs. Non-Smoker) ANOVA with all factors tested between-participants. There was a main effect of smoking status, *F*(1,786) = 79.70, *p* < 0.001, η^2^ = 0.092, such that smokers had less negative implicit smoking evaluations (*M* = -0.33, *SD* = 0.50) than did non-smokers (*M* = -0.67, *SD* = 0.44). There was also a main effect of condition, *F*(2,786) = 6.20, *p* = 0.002, η^2^ = 0.02 which was qualified by a significant interaction, *F*(2,786) = 10.82, *p* < 0.001, η^2^ = 0.03 (see **Figure 1**). In support of the hypothesis, implicit smoking evaluations of current smokers were more negative following an affective argument (*M* = -0.50, *SD* = 0.52) than following a cognitive argument (*M* = -0.11, *SD* = 0.47), *t*(91) = 3.77, *p* = 0.0003, *d* = 0.79, 95% CI_diff_ = 0.19, 0.60. In addition, implicit evaluations were marginally more negative following an affective argument than following a control message (*M* = -0.33, *SD* = 0.43), *t*(111) = 1.98, *p* = 0.063, *d* = 0.36, 95% CI_diff_ = -0.01, 0.35 while implicit evaluations were actually less anti-smoking following the cognitive message than following the control message, *t*(100) = 2.49, *p* = 0.015, *d* = 0.51, 95% CI_diff_ = -0.41, -0.05.

In contrast, for non-smokers, there was no observed difference in implicit evaluations depending on whether they read the affective persuasive message (*M* = -0.69, *SD* = 0.45) or the cognitive message (*M* = -0.74, *SD* = 0.36), *t*(420) = 1.19, *p* = 0.24, *d* = 0.12, 95% CI_diff_ = -0.13, 0.03. For non-smokers, implicit evaluations were more negative than the control message (*M* = -0.60, *SD* = 0.46) both after reading the affective message, *t*(433) = 2.18, *p* = 0.030, *d* = 0.20, 95% CI_diff_ = 0.01, 0.18 and after reading the cognitive message, *t*(417) = 3.53, *p* = 0.0005, *d* = 0.34, 95% CI_diff_ = 0.06, 0.22.

#### Explicit Evaluations

Explicit evaluations were also tested using a 3(Condition: Affect vs. Cognition vs. Control) × 2(Smoking Status: Smoker vs. Non-Smoker) ANOVA with all factors tested between-participants. There was a main effect of smoking status, *F*(1,888) = 905.90, *p* < 0.001, η^2^ = 0.51, such that smokers had less negative explicit smoking evaluations (*M* = -0.33, *SD* = 0.50) than did non-smokers (*M* = -0.67, *SD* = 0.44). There was also a main effect of condition, *F*(2,888) = 6.20, *p* = 0.004, η^2^ = 0.01 which was qualified by a significant interaction, *F*(2,888) = 5.78, *p* = 0.003, η^2^ = 0.01 (see **Figure 1**).

Self-reported smoking evaluations of current smokers were more negative following an affective argument (*M* = -0.10, *SD* = 1.85) than following a cognitive argument (*M* = 0.60, *SD* = 1.54), *t*(107) = 2.13, *p* = 0.036, *d* = 0.41, 95% CI_diff_ = 0.05, 1.36. Self-reported evaluations were marginally more negative following an affective argument than following a control message (*M* = 0.51, *SD* = 1.64), *t*(124) = 1.96, *p* = 0.052, *d* = 0.35, 95% CI_diff_ = -0.01, 1.33 while self-reported evaluations did not differ following the cognitive message compared to the control message, *t*(115) = 0.31, *p* = 0.76, *d* = 0.06, 95% CI_diff_ = -0.68, 0.50.

In contrast, for non-smokers, self-reported evaluations were marginally less negative after reading the affective message (*M* = -2.65, *SD* = 0.99) than the cognitive message (*M* = -2.79, *SD* = 0.72), *t*(467) = 1.81, *p* = 0.070, *d* = 0.17, 95% CI_diff_ = -0.30, 0.01. For non-smokers, explicit evaluations were not more negative after reading the affective message as compared to the control message (*M* = -2.49, *SD* = 1.23), *t*(485) = 1.55, *p* = 0.12, *d* = 0.14, 95% CI_diff_ = -0.04, 0.36 while reading the cognitive message did result in more negative explicit evaluations than reading the control message, *t*(478) = 3.26, *p* = 0.001, *d* = 0.30, 95% CI_diff_ = 0.12, 0.48.

### Discussion

In this third study, we went beyond the previous studies in several ways. First, we replicated the effects of verbal persuasive messages on implicit evaluations of smokers using yet another implicit measure (i.e., a standard IAT). Second, the inclusion of a control condition allowed us to conclude that an affective anti-smoking message increases the negativity of implicit evaluations of smoking in smokers, albeit only marginally. Interestingly, our study also shows that a cognitive anti-smoking message renders implicit evaluations in smokers less negative rather than more negative, thus showing evidence of reactance to the message. One possible explanation for this reactance effect is suggested by inoculation theory (e.g., McGuire, 1961). Specifically, smokers may be more commonly exposed to the type of information included in our cognitive message and are therefore more practiced at refuting this type of argument. Thus, when they read this type of messages, they may automatically engage counterarguments whose existence make implicit evaluations toward smoking less negative. Regardless of the validity of this explanation, the intriguing and unexpected reactance effect merits replication and further investigation.

On the one hand, the finding that the type of persuasive message matters for implicit evaluations is good news for persuasion researchers because it reveals that at least some (i.e., affective) persuasive messages can have desirable effects on implicit evaluations of important and well-known attitudes objects such as smoking. On the other hand, it shows that other (i.e., cognitive) persuasive messages can have an unexpected undesirable effect. Hence, persuasion researchers are well-advised to always check the effect of their messages on implicit evaluations.

We note that in Study 3, explicit evaluations of smokers were also more negative following the affective than cognitive message, while this was not true in any of the other four studies (including the footnoted study). To shed light on this ambiguity, we conducted a combined analysis across the five data collections detailed herein. This analysis did not reveal a main effect of message type (including only the affective and cognitive conditions) on explicit evaluations, *t*(858) = 3.01, *p* = 0.083, *d* = 0.12, suggesting that the messages had no consistent effect on explicit evaluations.

The lack of an effect on explicit evaluations in smokers lends credence to the argument that implicit evaluations of smoking in smokers are affective in nature and thus more susceptible to the impact of affective persuasive messages. On the one hand, it is possible that implicit evaluations of smoking are affective because they are implicit, that is, because all implicit evaluations may be primarily affective in nature. On the other hand, it is possible that implicit evaluations of smoking are affective because of the topic of smoking, that is, because all evaluations (i.e., implicit and explicit) of smoking in smokers are affective. The fact that only implicit but not explicit evaluations of smoking in smokers were influenced by the nature of the message is more in line with the first explanation. It should be noted, however, that the nature of the message had little impact on implicit evaluations of smoking in non-smokers. If implicit evaluations are by default affective in nature, affective messages should have had a bigger impact on implicit evaluations in non-smokers as well. Although speculative, the full set of results of Study 3 could be explained if one both assumes that (1) implicit evaluations are on average more affective in nature than explicit evaluations and (2) (implicit and explicit) evaluations of smokers are on average more affective in nature than (implicit and explicit) evaluations of non-smokers. Given that messages have the biggest effect when they match the affective or cognitive nature of the evaluations (Fabrigar and Petty, 1999), affective messages can be expected to have the biggest impact on implicit evaluations of smoking in smokers and the smallest impact on explicit evaluations of smoking in non-smokers whereas cognitive messages would have the biggest impact on explicit evaluations of smoking in non-smokers and the smallest effect (or even reversed effect) on implicit evaluations of smoking in smokers.

Our data are the first demonstration that persuasion of implicit evaluations depends on the affective or cognitive content of the persuasive message. Moreover, the differences in results between smokers and non-smokers show that one should consider not only the content of the message when attempting to change implicit evaluations but also the characteristics of the person whose implicit evaluations you are attempting to change. As such, our results provide important new information about persuasion of implicit evaluations.

## Study 4^^2^^

Until now, we have provided consistent evidence that an affective anti-smoking message is more effective than a cognitive anti-smoking message at impacting responses on implicit measures. Of particular note, we have done so using a variety of measures which differ from each other in a number of ways, thereby increasing the generalizability of our findings. A final concern is that the manipulations we used (taken from See et al., 2008) varied in a number of ways besides their observed ability to invoke feelings vs. thoughts. Although we can be sure that the content of the messages was crucial, we cannot be sure whether it is the affective or cognitive nature of the message that moderated implicit evaluations. We therefore ran a fourth study that allowed us to address this question. Following the manipulation and measurement, we asked participants to rate several properties of the messages. This allowed us to assess via mediational analyses which properties of the messages mediated the effect of the messages on implicit evaluations. Finally, we used the AMP as an index of implicit evaluations and included a control condition with the neutral message from Study 3.

### Method

#### Participants

Participants were 225 visitors to the Project Implicit research site who self-reported that they currently smoked cigarettes; 59.9% women (*M*_age_ = 29.8; *SD* = 11.2; range: 18–67). Participants were citizens of 36 different countries (58.4% USA, 7.7% UK, 5.0% Canada, all others < 3.6%). The average number of cigarettes smoked per day was 8.20 (*SD* = 7.60).

#### Materials

##### Persuasive messages

Affective, cognitive, and control messages were identical to those presented in previous studies.

##### Affect Misattribution Procedure

The AMP was identical to the one used in Study 1. Again, three separate scores were calculated, each being a proportion of times that participants responded “pleasant” following each of the three types of stimuli (i.e., smoking, non-smoking, and neutral). A difference score was then calculated by subtracting the scores for non-smoking from the scores for smoking. Thus, higher scores indicate more positive evaluations of smoking. Split-half reliability of the AMP scores was *r* = 0.60.

##### Explicit evaluations

Explicit evaluations were measured as in each of the previous studies with positive scores indicating a relative preference for smoking.

##### Questions about manipulation

Participants responded to five items about the persuasive argument. Three of the items consisted of a five-point scale anchored by 1 = “Not at all [relevant/strong/often]” and 5 = “Very [relevant/strong/often].” Specifically, we measured the relevance of the argument (“How relevant do you think the argument you just read is to your own smoking behavior?”), the strength of the argument (“How strong of an argument against smoking do you think it is?”), and how familiar participants would be with similar arguments (“How often do you hear arguments like this against smoking?”). In addition, they responded to the items, “How much would you say the message you read appeals to feelings and emotions?” and “How much would you say the message you read appeals to thoughts and beliefs?” using a 6-point scale where 1 = “Not at all” and 6 = “A lot.”

#### Procedure

The procedure largely followed that of Study 3. Participants assigned to this study were asked to self-report whether they currently smoked cigarettes. Those participants who indicated that they did not were reassigned to a new study, while those participants who indicated that they did smoke were asked to report how many cigarettes they currently smoke. Participants were then informed that they were completing a study about reading comprehension and memory before being assigned to read either the affective or cognitive persuasive anti-smoking message or a control message. Following the manipulation, participants completed an AMP and explicit evaluations in that order. Participants were then exposed to the message a second time to refamiliarize themselves with it before answering the three items about the message (relevance, strength, and familiarity) in that order, presented on a single page. They were then asked to report how much they believed the message appealed to their feelings and beliefs, with each question being presented on a separate page and in a random order.

### Results and Discussion

Overall, participants indicated an implicit preference for non-smoking relative to smoking when measured with the AMP, *M* = -0.16, *SD* = 0.29, *t*(222) = -7.91, *p* < 0.0001, *d* = -0.53. Participants’ also indicated a self-reported preference for non-smoking, *M* = -0.46, *SD* = 1.80, *t*(223) = -3.86, *p* = 0.0001, *d* = 0.26. Participants’ self-reported preferences were not reliably correlated with the AMP, *r* = 0.10, *p* = 0.12.

#### Explicit Evaluations

Smokers’ self-reported evaluations were unaffected by the type of persuasive message they read, *F*(2,221) = 0.44, *p* = 0.64. More specifically, explicit evaluations were comparable following the affective message (*M* = -0.57, *SD* = 1.74), cognitive message (*M* = -0.48, *SD* = 1.83), and control message (*M* = -0.30, *SD* = 1.86), all *t*s < 0.94, *p*s > 0.35, *d*s < 0.15.

#### Implicit Evaluations

We tested the effect of the persuasive messages on the AMP by comparing the AMP difference scores (i.e., difference in proportion of positive responses on smoking and non-smoking trials) between the three message conditions. AMP scores were significantly affected by the type of persuasive message, *F*(2,220) = 3.43, *p* = 0.034. Specifically, evaluations were more negative following the affective message (*M* = -0.21, *SD* = 0.33) than the cognitive message (*M* = -0.09, *SD* = 0.19), *t*(157) = -2.78, *p* = 0.006, *d* = 0.45, 95% CI_diff_ = 0.04, 0.21. The control message fell within these two means (*M* = -0.15, *SD* = 0.33), but did not differ significantly from either (both *t*s < 1.28, *p*s > 0.20, *d*s < 0.22).

#### Potential Mediators

Because our aim was to determine which aspects of the cognitive and affective message determined its impact on implicit evaluations, only the data of the affective and cognitive message conditions were included in the mediational analyses. In a confirmation of our pre-testing reported previously (see Study 1), the affective message was not seen as being stronger (*M* = 3.14, *SD* = 1.25) than the cognitive message (*M* = 3.38, *SD* = 1.10), *t*(157) = -1.30, *p* = 0.20, *d* = -0.21, thereby eliminating the strength of the persuasive message as a potential mediator of the observed effects. However, participants did report that they hear anti-smoking message like the cognitive one we used more often (*M* = 3.86, *SD* = 1.10) than the affective one (*M* = 2.88, *SD* = 1.36), *t*(157) = 4.94, *p* < 0.0001, *d* = 0.79. Perhaps, therefore, the effectiveness of the manipulation was due to this difference in novelty with participants paying more attention to the affective message and/or being more practiced at ignoring the cognitive message. However, the effect of the message type on AMP scores remained significant when controlling for how often participants reported being exposed to similar messages, *F*(1,156) = 6.05, *p* = 0.015, suggesting that the effect of the message is not explained via the affective message’s relative novelty.

Participants also reported that the cognitive anti-smoking message was more personally relevant (*M* = 3.22, *SD* = 1.23) than the affective one (*M* = 2.16, *SD* = 1.19), *t*(157) = 5.51, *p* < 0.0001, *d* = 0.87. While one might assume that personally relevant messages would be *more* impactful on implicit evaluations, it could be that smokers became more defensive and, for example, mentally counter-argued in the face of the more personally relevant cognitive message. However, after entering this variable into the regression equation, the effect of the message type remained significant, *F*(1,156) = 8.21, *p* = 0.005, suggesting that the effect of the message is also not explained by the higher personal relevance of the cognitive message relative to the affective message.

We hypothesized that our effects would be mediated by the perceived affective and cognitive nature of the messages. Reassuringly, participants in the affective condition were much more likely to agree that the persuasive message “appeals to feelings and emotions” (*M* = 4.48, *SD* = 1.50) than were participants in the cognitive condition (*M* = 2.84, *SD* = 1.55), *t*(157) = 6.76, *p* < 0.0001, *d* = 1.07. Conversely, participants in the cognitive condition were much more likely to agree that the persuasive message “appeals to rational thoughts and beliefs” (*M* = 4.29, *SD* = 1.50) than were participants in the affective condition (*M* = 3.44, *SD* = 1.52), *t*(158) = 3.55, *p* = 0.0005, *d* = 0.56. When entering ratings of how cognitive participants found the messages, the effect of the message type remained significant, *F*(1,156) = 8.10, *p* = 0.005. However, when entering ratings of how affective participants found the persuasive messages, the effect of the message type became non-significant, *F*(1,155) = 2.30, *p* = 0.13. The Sobel test for this mediational analyses was significant (*z* = 1.98, *p* = 0.048), supporting our assertion that the messages’ impact on implicit evaluations occurred via the feelings and emotions regarding smoking that participants experienced during the manipulations.

## General Discussion

In four studies, we demonstrated that persuasive messages influence even strongly held implicit evaluations in a manner that crucially depends on the content of the message. This was observed when the AMP, IAT, personalized IAT, and EP task were used to capture implicit evaluations. Our research therefore confirms that implicit evaluations can be changed not only by extended training methods that involve many trials (e.g., Baccus et al., 2004; Kawakami et al., 2007), but also by presenting a simple verbal message. Moreover, it demonstrates for the first time that persuasive messages can also change longstanding, strongly held evaluations such as evaluations of smoking.

The key result, however, concerned the impact of the type of message. It is important to remember that the two messages were rated as being equally strong in argument quality. Perhaps accordingly, both messages had a similar effect on explicit evaluations in smokers (i.e., the type of message did not moderate persuasion of explicit evaluations in smokers). Interestingly, whereas affective anti-smoking messages had the desired effect on the implicit evaluations of smoking in smokers, cognitive anti-smoking messages had an undesired effect in smokers (i.e., made the implicit evaluations of smoking more positive). In non-smokers, on the other hand, the nature of the message had little impact on implicit evaluations of smoking but tended to moderate changes on explicit evaluations. This pattern of results has a number of important implications. First, researchers can optimize the impact of persuasive messages on implicit evaluations by carefully considering the content of their message. More specifically, our data suggest that implicit evaluations that are affective in nature (e.g., implicit evaluations of smoking in smokers) are more susceptible to affective persuasive messages than to cognitive persuasive messages. Second, researchers who use persuasive messages to target explicit evaluations should also be aware of the effects of these messages on implicit evaluations. Although in many cases, both types of evaluations might be affected in the same way by persuasive messages, in some cases (e.g., when smokers are given cognitive anti-smoking messages), implicit evaluations could be influenced in ways that run counter to the intended effects. Some caution is required in drawing this conclusion, given that this unexpected result was significant only in Study 3, even though numerically a similar effect was found in Study 4.

In the current work, we were interested in examining whether persuasive messages influence implicit evaluations in a way that depends on the content of the message. Our primary focus was not on the mechanisms by which or reasons for which these changes occur. Nevertheless, we do have clear hypotheses about these issues. First, the fact that affective messages had stronger desired effects on implicit evaluations of smoking in smokers is most likely driven by the general principle that persuasive messages are more effective when they match the to-be-changed evaluations (e.g., Fabrigar and Petty, 1999). As argued in the introduction, implicit evaluations of smoking in smokers are most likely affect-based because of their implicit nature and the fact that evaluations in smokers generally tend to be affect-based (Trafimow and Sheeran, 1998). Although it is not entirely clear which of these two elements (i.e., the implicit nature or the smoking-related nature) is crucial in this respect (see discussion of Study 3), the fact that affective messages had the biggest desired impact on implicit evaluations of smoking in smokers is most likely due to the match between the content of the message and targeted evaluations. Additionally, in Study 4, the mediational analysis provided evidence that the observed effects of the persuasive messages occurred via the extent to which participants viewed the persuasive messages as being relevant to their emotions and feelings regarding smoking.

Second, the contrast effect observed in Study 3 of the cognitive message on implicit evaluations of smoking in smokers could be seen as an instance of reactance. This would entail that smokers had a negative reaction toward the cognitive message which engendered a positive affective reaction toward smoking. Such an idea is in line with inoculation theory (McGuire, 1961) which implies that smokers may automatically generate counterarguments to well-known cognitive arguments. These counterarguments may be affective in nature or generate positive feelings toward smoking. Regardless of the merits of this *post hoc* explanation, the reactance effect of cognitive anti-smoking messages observed in Study 3 is an intriguing and unexpected finding that deserves further investigation. Also as noted previously, although this result was in the same direction using the AMP (in Study 4), it did not reach significance. We feel this effect is one that deserves future studies more specifically focused on this issue.

Third, as discussed previously, several mental processes could be involved in the effect of verbal messages on implicit evaluations. According to the APE model (Gawronski and Bodenhausen, 2011), for instance, implicit evaluations can be changed directly by changing underlying associations or by changing which associations are activated. One way future research might distinguish between these two possibilities is by examining how long effects of persuasive messages on implicit evaluations last. To date, very little is known about the longevity of changes in implicit attitudes, including changes induced by training procedures (but see Ranganath and Nosek, 2008; Wiers et al., 2011; Smith et al., 2012; Mann and Ferguson, 2015).

It may also be informative to test the efficacy of the types of verbal appeals used in the current work against the longer training paradigms commonly utilized. These two types of manipulations could have more in common than seems likely on the surface. It is possible, for example, that training techniques commonly used to change implicit evaluations exert their influence indirectly through leading participants to construct explanations for their behavior which then act as persuasive messages. For instance, implicit attitudes toward smoking might become more negative in a participant tasked with pushing away images of cigarettes (see Wiers et al., 2011) because the participant forms negative conscious beliefs about smoking on the basis of the training (e.g., “I push away cigarettes because they are bad”).

Although the current work was not designed to interpret null effects, issues of power are still relevant. The current samples were appropriately sized to test for medium-sized effects (*post hoc* power analyses indicate that all comparisons had >80% power, except for the comparison between affect and cognition in Study 3 for smokers, which had 73% power). That said, the studies were still not well-powered for finding small effects (even the largest samples of Study 2 had less than 40% power to detect such effects). As such, larger samples will be useful in future work, especially in understanding potential effects on explicit measures.

### Limitations and Future Directions

The current work had a number of limitations, some of which may inspire future investigations. The most obvious room for extension of the current work is regarding the attitude object and the manipulation. We chose to focus the breadth of our investigation on whether a single manipulation could change a number of measures of implicit evaluations of smoking. As such, we focused on fairly direct replications. However, future work looking at different attitude objects (e.g., drinking alcohol) and using a number of different methods of manipulating affect (and, potentially, cognition) will help to understand whether the effect generalizes beyond the current materials. This would also aid in differentiating between questions we raised previously as to whether affective messages are best at changing implicit evaluations only when the attitude object itself is relatively affective in nature (e.g., smokers’ evaluations of smoking).

Additionally, the observation that smokers’ implicit evaluations of smoking are negative may seem counterintuitive, given that they habitually smoke and that previous research has demonstrated that implicit measures are more related to impulsive behavior than to relatively reflective behavior (e.g., Hofmann et al., 2008; Friese and Hofmann, 2009). In other words, given that smokers smoke, their gut-level responses must be positive, otherwise why do they smoke? Although this could cast doubt on the fitness of our implicit measures, we note that the findings regarding the direction of our implicit measures is in line with a number of previous investigations, to which the reader interested in why implicit smoking evaluations may be negative is directed (e.g., Swanson et al., 2001; Sherman et al., 2003; Bassett and Dabbs, 2005; Huijding et al., 2005; Payne et al., 2007; Rudman et al., 2007; but see De Houwer et al., 2007). It is important, however, to bear in mind that our research was concerned with novel ways of changing implicit evaluations rather than establishing the absolute value or zero-point of implicit evaluations.

Another limitation is that, although smokers’ implicit evaluations after reading the control message always fell in between implicit evaluations after reading the affective and cognitive messages, the pattern of significance was inconsistent. Future work with a higher-powered design would help to nail this effect down. Relatedly, aspects of the control condition could be changed. One option is simply to include a measurement condition with no reading involved, which would then act more as a baseline condition. Additionally, we wrote the current control message to be negative, given that the persuasive anti-smoking messages were negative in tone. However, this may have had the effect of priming negativity and led to more negative implicit smoking evaluations as compared to baseline evaluations. If that is true, a control condition which is more positive in tone may be more reliably distinguishable from the affective condition and may remove the undesired effect of cognitive messages. Please note, however, that our results have merit irrespective of the outcome of this future research. First, persuasive messages will remain an essential tool in the fight against smoking. In this applied context, control messages that are unrelated to smoking of course have little value. Instead, only anti-smoking messages will be used. Whenever these anti-smoking messages are used, a decision has to be made about how much emphasis is put on affective or cognitive arguments. Our work provides important new information for making that decision: everything else being equal, affective messages have more desirable effects on implicit evaluations than cognitive messages. Second, we want to repeat the fact that our results also have more general implications. As we noted above, they show for the first time that researchers can optimize the impact of persuasive messages on implicit evaluations by carefully considering the content of their message.

## Conclusion

In sum, our studies show that persuasive messages can be used to change long-standing implicit evaluations when the content of the messages is specifically geared toward changing implicit evaluations in a specific target population. We showed that persuasive messages can influence implicit evaluations in a way depends upon the specific message content and properties of the person who is exposed to the message and that this effect is mediated by feelings and emotions. Our results contribute not only to our understanding of persuasion and implicit evaluation, but also have implications for research on smoking and substance use more generally. Although we did not study smoking behavior as such, it is widely assumed that implicit evaluations of smoking have an impact on (smoking) behavior (e.g., Roefs et al., 2011). Hence, our results open up new avenues for understanding and changing smoking behavior. For instance, it suggests that cognitive anti-smoking messages might actually increase smoking in smokers (see van ‘t Riet and Ruiter, 2011, for a review) because these messages render smokers’ implicit evaluations of smoking less negative. Whether persuasion-induced changes in implicit evaluations of smoking actually influence behavior, however, needs to be addressed in future research. In sum, the current work adds to the overwhelming evidence that implicit evaluations can be changed, contributes to the very recent assertion that they can be changed via direct persuasive messages, and is the first to show that the content of such persuasive messages is integral for understanding the direction of changes in implicit evaluations.

## Conflict of Interest Statement

The authors declare that the research was conducted in the absence of any commercial or financial relationships that could be construed as a potential conflict of interest.

## Footnotes

1 We chose our sample sizes *a priori*, though somewhat arbitrarily, aiming for roughly 200 smokers per study. When sample sizes were near that number, we requested that the study be removed from the Project Implicit research pool, which takes a few days to occur. Variation in the number of participants across studies is due to lag time in either checking the study n or in the study coming down. The number of smokers in Study 3 is somewhat lower than hoped for as we had to estimate the proportion of smokers within the sample based on previous data collections at Project Implicit. The number of completed sessions for a study can be checked without downloading raw data and at no point did we look at data until the study removal request was made and the study was completed.

2 We also ran a study (*n* = 122) identical to Study 4 (without the control condition) using an IAT. We did not observe an effect of condition; IAT scores equally negative following the affective message (*M* = -0.47, *SD* = 0.52) and the cognitive message (*M* = -0.54, *SD* = 0.52), *t*(108) = 0.71, *p* = 0.48, *d* = -0.14, 95% CI_diff_ = -0.27, 0.13. We also did not observe a significant effect of the messages on explicit evaluations; evaluations were similarly negative following the affective message (*M* = -0.39, *SD* = 2.02) and the cognitive message (*M* = 0.01, *SD* = 1.92), *t*(119) = -1.12, *p* = 0.26, *d* = 0.20, 95% CI_diff_ = -0.31, 1.12. When combining the results of this study and those of Study 3 – which also employed an IAT – the effect of message type did not reach conventional levels of significance, *t*(201) = 1.62, *p* = 0.11, *d* = 0.23, although the means were in the hypothesized direction. Thus, one could conclude that IAT effects are not particularly sensitive to the content of persuasive messages. However, the different studies were not run at similar times of the year (e.g., only the additional study that failed to reveal the expected effect was run during the summer holidays) and given that in both of the IAT studies, the effect of message content was in the expected direction, we are hesitant to draw strong conclusions regarding this issue.
