# Supplementary material for: Hooked on a feeling: affective anti-smoking messages are more effective than cognitive messages at changing implicit evaluations of smoking
Source: Front Psychol. 2015 Oct 6;6:1488. doi: 10.3389/fpsyg.2015.01488 (PMC4617384; doi:10.3389/fpsyg.2015.01488)
Supplement: Supplementary file 1 [file Data_Sheet_1.DOCX]

**Appendix**

Affective Message

John tried his first cigarette at the age of 14. Smoking was a means for him to fit in with his new friends at school. It did not take long for John to realize that he was addicted to cigarettes. His addiction turned him into a sneak, a liar, and a thief. He stole money from his younger brother to buy cigarettes because he could not afford to buy the quantity he had to smoke. Even after he became a father to two children, the lying did not stop. He borrowed money from his brother and lied that the money was for an X-Box for his son's birthday.
He lied to his doctor, insisting that he smoked "only less than a pack a day." He was absolutely ashamed but could not stop. He was compelled to such action, all because of his nicotine addiction.
 In 1999, John was diagnosed with cancer in the larynx. The doctor suggested a laryngectomy, which meant that John could not talk again. When John first got home after the operation, his children ran away from him when they saw the hole in his throat. He opened his mouth to call them, but no voice came out. Soon later, he suffered from withdrawal symptoms from having stopped smoking. He experienced increased anger and hostility and had problems with basic cognitive functions such as language comprehension. Worse still, his older son, Jimmy, was diagnosed with bronchitis, a disease common in people exposed to secondhand smoke. Guilt overcame John, but it was too late.
 When smoking doesn't kill, it destroys.

Cognitive Message

Forty years have passed since the first Surgeon General's report on smoking and health. Yet smoking remains the leading preventable cause of death in this country. Tobacco continues to cost our society too many lives and too many dollars. The new Surgeon General's report illustrates the harmful impact of smoking on many organs in the body. The report's statistics and conclusions highlight the necessity of remaining vigilant in our smoking prevention efforts.
 For example, smoking causes 87% of lung cancer, and most cancers of the larynx, oral cavity, esophagus, and bladder. In addition, secondhand smoke is responsible for an estimated 3,000 lung cancer deaths among nonsmokers each year. This is no surprise considering that tobacco smoke contains thousands of chemical agents, including over 60 substances that are known to cause cancer.
 Cigarette smoking has been the most popular method of taking nicotine since the beginning of the 20th century. Nicotine is highly addictive. Nicotine provides an almost immediate "kick" because it causes a discharge of epinephrine from the adrenal cortex.
This "kick" then leads the abuser to seek more nicotine. Addiction to nicotine results in withdrawal symptoms when a person tries to stop smoking. For example, during periods of abstinence and/or craving, smokers have shown impairment across a wide range of psychomotor and cognitive functions, such as language comprehension.
 Such are the harmful effects of smoking.

Control Message

Deer Springs is an apartment complex that has seen better days. It was built quickly during a period of growth in the area in the late 1980s and hasn't been upgraded at all since then. While it may have been nice when it was built, the vinyl siding is now starting to crack and many of the sidewalks leading to the units are completely broken up.

Also, many residents complain about parking. The area where Deer Springs is located is full of apartment complexes and there is quite a fight for parking spaces. Deer Springs has no reserved parking because the street is owned by the city. Therefore it's public parking. You just have to get home early or you can't park your car without a lot of trouble.

Many of the residents of Deer Springs seem to have no idea how to live respectfully in a multi-unit apartment complex. It is very common to see a dog barking on the balcony and many people leave their windows open with their televisions playing very loudly. As a result, getting to sleep before 11pm can be quite difficult!

In addition to major renovations, Deer Springs would be much better if there were 5 to 10 security guards patrolling the area. Over the years, the area of town where Deer Springs is located has gotten less safe, and the presence of gates or security guards would likely make the residents feel safer.

In short, Deer Springs Apartment Complex is not a very nice place to live.
